# Supplementary material for: The final spawning ground of Tachypleus gigas (Müller, 1785) on the east Peninsular Malaysia is at risk: a call for action
Source: PeerJ. 2016 Jul 19;4:e2232. doi: 10.7717/peerj.2232 (PMC4957986; doi:10.7717/peerj.2232)
Supplement: Data S1 [file peerj-04-2232-s001.pdf]

Table 1: Complete eco-biological observations from the *Tachypleus gigas* nesting sites (1-3) during the first series (2009-2010) of new moon surveys at Pantai Balok

		2009															2010											
		August			September			October			November			December			January			February			March			April		
		S1	S2	S3	S1	S2	S3	S1	S2	S3	S1	S2	S3	S1	S2	S3	S1	S2	S3	S1	S2	S3	S1	S2	S3	S1	S2	S3
Biology	Nest (nos.)	-	1	-	-	-	-	2	-	-	1	-	-	-	-	-	-	-	-	-	-	-	2	-	-	2	-	-
	Egg (nos.)	-	104	-	-	-	-	385	-	-	92	-	-	-	-	-	-	-	-	-	-	-	158	-	-	320	-	-
	Clutches (nos.)	-	1	-	-	-	-	3	-	-	1	-	-	-	-	-	-	-	-	-	-	-	2	-	-	3	-	-
	Male (nos.)	-	2	1	-	-	-	-	-	-	1	-	-	-	-	-	-	-	-	-	-	-	1	4	1	-	2	1
	Female (nos.)	2	-	-	-	-	-	-	-	-	2	-	-	-	-	-	-	-	-	-	-	-	-	1	4	3	-	-
Sediment	Mean (*X*φ)	1.9	1.3	1.3	1.7	1.4	1.8	1.2	2.1	1.7	1.2	2.4	1.9	1.4	2.4	2.0	1.7	2.1	2.5	1.4	2.1	2.4	1.1	2.1	2.2	1.1	1.9	1.9
	Sorting (*σ*φ)	1.0	1.0	1.2	1.1	1.0	1.3	1.0	0.7	1.4	0.9	0.6	1.5	0.9	0.5	1.7	1.3	0.6	1.2	1.1	0.9	1.0	1.1	0.8	1.1	1.1	1.0	1.0
	Skewness (*SK*φ)	0.3	0.1	0.0	0.1	0.1	0.1	0.1	0.3	0.3	0.0	0.2	0.5	0.0	0.4	0.7	0.1	0.3	1.0	0.0	0.2	0.8	0.0	0.1	0.5	0.0	0.1	0.5
	Kurtosis (*K*φ)	3.1	3.3	2.3	3.0	3.6	2.3	2.7	4.0	2.3	2.4	4.5	2.3	2.2	3.9	2.1	2.6	4.1	2.4	2.7	4.0	2.7	2.7	3.7	2.9	3.1	3.4	3.3
	Gravel (%)	2.0	2.4	2.2	2.3	2.6	1.5	2.8	0.4	0.8	2.8	0.1	0.4	1.4	0.3	0.5	1.8	0.6	1.2	2.0	1.7	0.8	2.3	0.4	2.0	1.9	0.5	2.8
	Sand (%)	93.2	95.9	94.8	93.2	95.4	95.1	93.5	98.4	95.0	93.9	99.6	94.8	94.7	99.5	93.6	95.2	99.0	94.3	95.2	97.5	95.3	94.9	97.9	94.1	94.6	96.9	93.1
	Silt & Clay (%)	4.8	1.8	3.0	4.5	2.0	3.4	3.7	1.2	4.2	3.3	0.3	4.7	3.8	0.3	5.9	3.0	0.4	4.5	2.8	0.8	4.0	2.9	1.7	3.9	3.5	2.6	4.2
	0.125 mm (%)	4.1	1.8	2.1	2.9	2.8	2.9	2.3	5.4	3.7	1.8	5.8	4.2	2.0	5.7	4.8	2.3	4.9	4.0	2.0	3.7	3.7	1.6	3.3	2.8	2.7	4.2	3.0
	0.180 mm (%)	15.3	10.0	11.4	14.6	11.9	12.4	13.7	14.6	14.7	11.9	15.8	16.0	12.5	15.9	16.5	14.2	15.4	15.5	13.2	14.7	14.8	11.8	14.0	14.1	11.9	14.7	13.0
	0.250 mm (%)	21.9	19.4	19.7	21.1	19.1	21.5	20.0	20.2	23.9	19.7	21.5	26.3	19.8	22.9	28.8	21.8	26.4	24.5	21.0	24.8	23.0	19.6	23.2	21.6	18.5	20.4	18.7
	Moisture depth (cm)	3.7	5.1	4.5	4.0	3.9	4.0	3.7	3.0	3.1	3.1	2.3	2.3	2.8	2.5	2.7	3.8	4.1	4.7	4.6	5.1	5.6	5.9	5.9	6.3	6.2	6.5	6.5
	Temperature (°C)	32.6	32.4	32.5	31.5	31.6	31.7	30.0	30.2	30.5	29.3	29.3	29.5	28.5	28.7	28.9	27.2	27.4	27.8	28.8	28.5	28.7	29.9	30.0	30.2	32.3	32.3	32.5
	pH	6.2	6.2	6.1	6.4	6.3	6.1	6.2	6.3	6.4	6.5	6.7	6.7	6.7	6.6	6.7	6.7	6.5	6.3	6.4	6.3	6.1	6.0	5.8	5.9	5.5	5.4	5.3
Water	Temperature (°C)	29.7	29.6	29.5	29.7	29.4	29.2	31.1	31.3	31.3	31.0	31.2	31.4	27.7	27.8	27.8	26.8	27.2	27.4	28.4	28.6	28.9	30.3	30.2	30.3	31.6	31.3	31.0
	pH	7.7	7.6	7.7	7.5	7.5	7.6	7.6	7.2	7.6	8.0	7.4	7.7	8.0	7.9	7.8	7.5	7.6	7.4	7.2	7.3	7.0	7.0	7.2	6.8	7.1	7.5	7.3
	Salinity (‰)	30.6	32.3	32.1	34.7	34.6	32.1	35.4	37.1	31.9	32.9	32.1	30.2	31.6	28.9	30.2	34.6	34.4	34.9	36.5	37.9	38.4	35.9	38.6	39.4	36.6	35.4	36.7
	DO (mg l^{-1})	4.8	5.2	5.5	4.2	5.1	5.2	4.6	4.2	5.0	5.6	4.2	5.8	6.7	6.1	6.7	6.6	6.4	6.9	6.1	5.6	6.4	5.7	5.5	6.0	5.9	6.0	5.7

'-' no sample availability during the investigation period

Table 2: Complete eco-biological observations from the *Tachypleus gigas* nesting sites (1-3) during the first series (2009-2010) of full moon surveys at Pantai Balok

		2009																		2010											
		July			August			September			October			November			December			January			February			March			April		
		S1	S2	S3	S1	S2	S3	S1	S2	S3	S1	S2	S3	S1	S2	S3	S1	S2	S3	S1	S2	S3	S1	S2	S3	S1	S2	S3	S1	S2	S3
Biology	Nest (nos.)	-	-	3	-	3	-	-	-	-	2	-	-	1	-	-	-	-	-	-	-	-	-	-	-	2	-	-	4	-	-
	Egg (nos.)	-	-	502	-	318	-	-	-	-	340	-	-	114	-	-	-	-	-	-	-	-	-	-	-	237	-	-	455	-	-
	Clutches (nos.)	-	-	4	-	3	-	-	-	-	2	-	-	1	-	-	-	-	-	-	-	-	-	-	-	3	-	-	5	-	-
	Male (nos.)	1	4	-	-	1	2	-	-	-	1	2	-	2	-	-	-	-	-	-	-	-	-	-	-	1	1	-	1	2	-
	Female (nos.)	-	2	4	2	2	4	-	-	-	4	-	-	2	1	-	-	-	-	-	-	-	-	-	-	2	-	-	-	-	3
Sediment	Mean ($X\varphi$)	2.1	1.4	1.1	1.8	1.1	1.5	1.5	1.7	1.9	1.1	2.4	1.7	1.2	2.5	2.0	1.6	2.4	2.1	1.9	2.1	2.4	1.5	2.0	2.6	1.2	2.1	2.4	1.0	2.1	2.1
	Sorting ($\sigma\varphi$)	1.0	0.9	1.1	1.0	1.0	1.2	1.1	0.9	1.4	0.9	0.5	1.4	0.8	0.7	1.7	0.9	0.4	1.5	1.0	0.6	1.3	1.1	0.8	1.1	1.0	0.9	0.9	1.0	1.0	1.1
	Skewness ($SK\varphi$)	0.5	0.5	-0.1	0.2	0.0	0.0	0.1	0.3	0.2	0.0	0.1	0.3	0.0	0.2	0.6	0.1	0.6	0.9	0.1	0.4	1.0	0.1	0.2	0.9	0.0	0.1	0.7	0.0	0.1	0.4
	Kurtosis ($K\varphi$)	3.3	2.5	2.5	3.0	3.9	2.3	2.9	3.3	2.2	2.5	4.6	2.4	2.1	4.4	2.2	2.3	3.4	2.1	2.4	4.1	2.4	2.9	4.1	2.4	2.6	3.9	2.7	2.7	3.7	3.1
	Gravel (%)	1.3	0.8	2.4	2.6	2.8	1.8	2.2	1.0	1.1	2.8	0.0	0.5	2.2	0.1	0.4	0.2	0.5	0.8	1.2	0.1	1.0	2.1	0.1	1.4	2.4	0.4	1.9	2.1	0.4	2.3
	Sand (%)	93.6	97.1	94.7	92.8	94.6	95.1	93.7	97.3	95.3	93.7	99.5	95.2	94.6	99.7	94.5	95.7	99.2	93.0	95.3	99.7	93.2	95.4	99.3	95.0	94.6	98.4	94.0	94.7	97.5	94.1
	Silt & Clay (%)	5.1	2.1	2.9	4.7	2.6	3.1	4.2	1.8	3.5	3.5	0.5	4.2	3.3	0.1	5.1	4.1	0.3	6.2	3.6	0.2	5.7	2.6	0.6	3.6	3.0	1.1	4.2	3.2	2.2	3.6
	0.125 mm (%)	4.7	2.2	1.7	3.2	1.4	2.4	2.5	4.5	3.1	1.9	6.0	4.1	1.9	5.3	4.3	2.3	6.1	5.2	2.6	5.1	4.1	2.2	4.8	3.8	1.9	3.2	3.2	1.7	3.6	2.7
	0.180 mm (%)	16.1	9.8	11.4	14.6	10.6	11.3	14.6	13.6	13.1	12.3	15.6	15.6	11.6	16.0	16.4	13.5	15.6	16.8	14.7	15.9	15.8	13.8	14.4	15.3	12.4	14.9	14.6	11.4	13.4	13.7
	0.250 mm (%)	22.3	20.2	19.5	21.7	19.0	20.1	20.4	19.6	22.4	19.5	20.7	25.8	19.6	22.5	27.2	20.1	23.2	29.8	22.3	27.2	25.5	21.3	25.6	23.1	19.7	24.5	22.7	19.5	22.7	20.3
	Moisture depth (cm)	3.2	5.7	4.8	4.1	4.6	4.3	3.9	3.2	3.8	3.5	2.8	2.4	2.7	1.9	2.2	2.8	3.1	3.2	3.4	3.5	4.1	4.2	4.7	5.3	5.1	5.6	5.9	6.7	6.3	6.8
	Temperature (°C)	32.2	31.2	32.7	30.8	31.2	31.5	30.3	30.6	30.1	30.1	29.9	29.9	28.7	29.1	28.8	28.7	28.7	28.8	27.2	27.6	27.7	28.1	27.9	28.2	30.2	30.8	30.6	32.5	32.0	32.5
	pH	6.1	6.1	5.9	6.2	6.5	6.5	6.8	6.3	6.6	6.5	6.6	6.6	6.3	6.1	6.4	6.7	6.4	6.3	6.1	6.2	6.3	6.2	6.1	6.1	5.8	5.9	5.8	5.8	5.2	5.5
Water	Temperature (°C)	29.4	29.7	29.5	30.0	29.6	29.5	29.3	29.3	29.0	32.9	33.2	33.4	29.3	29.4	29.5	26.2	26.4	26.2	26.3	26.8	27.0	27.4	27.6	27.8	29.5	29.7	30.1	31.2	30.8	30.5
	pH	7.8	7.5	7.5	7.7	7.7	7.8	7.4	7.4	7.5	7.8	7.1	7.6	8.2	7.6	7.7	7.9	8.1	7.8	7.6	7.8	7.5	7.4	7.4	7.2	6.9	7.1	6.7	7.2	7.3	7.0
	Salinity (‰)	28.7	31.0	32.1	32.5	33.5	32.0	36.7	35.7	32.1	34.2	38.4	31.8	31.7	26.4	28.8	31.5	31.3	31.4	33.5	32.2	33.2	35.8	36.7	36.6	37.2	39.2	40.4	34.6	37.8	38.5
	DO (mg l^{-1})	5.4	5.1	5.0	4.3	5.4	6.0	4.2	4.9	4.5	5.0	3.5	5.5	6.2	4.9	6.0	7.2	7.1	7.3	6.8	6.9	7.1	6.3	5.9	6.7	5.8	5.2	6.1	5.6	5.7	6.0

'-' no sample availability during the investigation period

Table 3: Complete eco-biological observations from the *Tachypleus gigas* nesting sites (1-3) during the second series (2010) of new moon surveys at Pantai Balok

		2010																				
		June			July			August			September			October			November			December		
		S1	S2	S3	S1	S2	S3	S1	S2	S3	S1	S2	S3	S1	S2	S3	S1	S2	S3	S1	S2	S3
Biology	Nest (nos.)	-	-	-	-	-	2	-	2	-	-	-	-	-	-	-	-	-	-	-	-	-
	Egg (nos.)	-	-	-	-	-	581	-	455	-	-	-	-	-	-	-	-	-	-	-	-	-
	Clutches (nos.)	-	-	-	-	-	3	-	2	-	-	-	-	-	-	-	-	-	-	-	-	-
	Male (nos.)	-	1	1	-	3	-	-	2	-	-	-	-	-	1	-	-	-	-	-	-	-
	Female (nos.)	-	1	1	-	2	-	-	1	-	-	-	-	-	-	-	-	-	-	-	-	-
Sediment	Mean ($X\varphi$)	1.9	1.8	1.9	1.7	1.7	2.1	1.2	1.6	0.6	0.7	0.5	1.0	0.0	0.0	0.0	1.6	2.4	0.7	2.0	1.9	1.2
	Sorting ($\sigma\varphi$)	1.1	1.1	1.1	1.0	1.0	0.9	1.4	1.1	1.3	1.4	1.3	1.6	0.5	0.5	0.6	1.1	1.0	1.1	1.2	0.9	0.9
	Skewness ($SK\varphi$)	-1.7	-1.6	-1.6	-1.1	-0.7	-1.8	-0.1	-0.7	0.5	0.1	0.4	-0.1	1.4	0.9	2.1	-1.1	-2.2	0.0	-0.9	-0.9	-0.5
	Kurtosis ($K\varphi$)	5.5	5.1	5.3	4.5	3.9	6.8	3.5	3.6	2.5	1.8	2.1	1.5	6.5	4.9	8.8	5.9	8.9	2.5	4.7	4.0	3.1
	Gravel (%)	5.0	5.2	4.3	3.1	1.9	1.4	7.7	3.2	10.2	4.6	4.3	4.8	0.6	0.7	0.5	7.1	4.1	9.8	2.2	1.0	2.5
	Sand (%)	94.3	94.3	95.0	95.5	96.0	97.2	88.7	93.5	86.8	94.9	95.1	94.9	99.4	99.3	99.5	91.9	94.8	89.5	97.6	99.0	97.3
	Silt & Clay (%)	0.6	0.5	0.7	1.4	2.1	1.4	3.6	3.3	3.0	0.5	0.6	0.3	0.0	0.0	0.0	1.0	1.2	0.7	0.2	0.1	0.3
	0.125 mm (%)	18.8	17.1	18.5	13.9	9.6	11.9	5.9	7.7	2.6	5.0	2.1	7.9	0.1	0.1	0.2	17.7	34.1	0.5	14.5	21.0	1.0
	0.180 mm (%)	28.5	26.1	27.8	28.4	29.6	60.5	25.7	26.3	18.6	22.1	15.6	28.1	0.3	0.2	0.6	18.0	21.5	13.5	26.7	21.0	19.8
	0.250 mm (%)	41.3	40.5	37.7	26.5	15.9	7.4	14.8	16.5	9.3	5.3	5.3	5.2	2.0	0.3	3.9	10.5	11.8	8.7	30.2	28.9	17.0
	Moisture depth (cm)	6.3	5.2	6.7	4.3	4.8	6.1	3.6	4.4	5.7	3.2	4.5	5.3	3.7	4.1	5.2	4.2	4.8	4.6	3.9	4.5	4.8
	Temperature (°C)	34.4	32.3	32.7	32.5	31.8	31.6	32.8	32.4	32.2	33.2	33.5	33.0	31.3	30.7	31.2	29.4	29.5	29.3	27.2	27.4	27.6
	pH	6.6	6.2	6.3	6.1	6.1	6.0	6.5	6.3	6.5	6.5	6.6	6.5	6.4	6.4	6.4	6.2	6.3	6.2	6.6	6.4	6.5
	Total Organic Carbon (%)	0.2	0.2	0.2	0.1	0.1	0.2	0.1	0.1	0.1	0.3	0.3	0.3	0.2	0.2	0.2	0.2	0.1	0.2	0.2	0.1	0.2
	Cd^{2+} (mg kg^{-1})	4.6	3.3	5.4	4.4	3.6	4.0	3.9	3.6	3.3	1.5	1.4	1.6	1.9	2.5	1.5	3.7	4.1	3.1	5.5	4.0	4.4
	Cr^{3+} (mg kg^{-1})	30.9	27.4	31.0	29.0	27.6	27.0	26.7	24.0	22.7	45.3	41.9	47.6	19.9	21.5	20.6	28.7	31.2	24.7	26.0	20.4	19.1
	Cu^{2+} (mg kg^{-1})	6.6	8.7	3.8	6.2	8.8	8.9	17.3	15.0	15.3	10.1	8.9	11.0	7.1	7.2	7.7	8.6	10.9	5.9	1.0	1.0	0.4
	Pb^{2+} (mg kg^{-1})	5.2	4.6	5.1	6.3	7.6	9.1	10.4	9.3	8.9	7.5	7.4	7.5	7.6	8.4	7.7	9.1	8.3	9.5	14.2	11.8	9.9
	Se^{2+} (mg kg^{-1})	9.8	8.3	10.3	8.1	6.0	9.3	14.4	11.2	13.9	9.1	9.4	8.7	8.3	8.6	8.9	9.2	10.0	8.0	14.1	8.0	13.5
	Zn^{2+} (mg kg^{-1})	32.8	29.5	32.6	27.2	22.5	23.9	32.2	27.5	28.7	24.1	23.3	24.3	18.0	18.7	19.3	30.9	32.2	28.0	20.5	16.3	14.8
Water	Temperature (°C)	29.4	29.5	29.5	29.5	29.6	29.5	30.0	30.0	29.8	31.6	31.8	31.7	29.8	29.6	29.6	27.2	27.4	27.4	26.2	26.4	26.4
	pH	7.8	7.5	7.2	7.3	7.6	7.5	7.6	7.8	7.4	7.3	7.1	7.5	7.1	7.5	7.1	8.0	7.8	7.6	7.0	7.3	7.5
	Salinity (‰)	32.1	32.8	33.6	32.0	32.8	33.6	32.5	32.9	33.3	32.1	32.7	33.1	6.6	10.7	12.0	31.3	32.5	34.7	0.5	1.4	2.1
	Dissolved Oxygen (mg l^{-1})	5.4	5.3	5.3	6.0	5.9	5.8	4.3	4.7	4.8	4.0	4.0	4.2	4.0	3.7	3.4	7.1	7.2	7.2	5.3	5.5	5.6
	NO_2^- (mg l^{-1})	0.0	0.0	0.0	0.1	0.1	0.1	0.1	0.1	0.1	0.1	0.1	0.1	0.0	0.0	0.0	0.0	0.0	0.0	0.0	0.0	0.0
	NO_3^- (mg l^{-1})	0.9	1.3	1.4	1.4	1.6	1.5	1.1	1.1	0.8	1.3	1.3	1.4	0.3	0.6	0.3	0.8	0.7	0.9	1.2	1.0	1.2
	PO_4^{3-} (mg l^{-1})	0.3	0.3	0.3	0.3	0.3	0.3	0.4	0.4	0.4	0.4	0.4	0.4	0.3	0.3	0.4	0.3	0.4	0.4	0.4	0.4	0.4
	S^{2-} (µg l^{-1})	1.3	1.7	1.0	4.0	4.7	6.0	11.7	7.0	12.7	2.7	5.0	1.7	15.0	9.0	12.7	25.0	23.0	17.7	10.0	7.7	8.3
	Chl-*a* (mg l^{-1})	0.1	0.1	0.1	0.1	0.1	0.1	0.0	0.0	0.0	0.1	0.1	0.5	0.1	0.1	0.1	0.1	0.1	0.1	0.0	0.0	0.0

'-' no sample availability during the investigation period

Table 4: Complete eco-biological observations from the *Tachypleus gigas* nesting sites (1-3) during the second series (2011) of full moon surveys at Pantai Balok

		2011																	
		January			February			March			April			May			June		
		S1	S2	S3	S1	S2	S3	S1	S2	S3	S1	S2	S3	S1	S2	S3	S1	S2	S3
Biology	Nest (nos.)	-	-	-	-	-	-	3	-	-	1	-	1	-	-	-	-	-	-
	Egg (nos.)	-	-	-	-	-	-	556	-	-	342	-	18	-	-	-	-	-	-
	Clutches (nos.)	-	-	-	-	-	-	3	-	-	1	-	1	-	-	-	-	-	-
	Male (nos.)	-	-	-	-	-	-	2	-	-	1	-	-	-	-	1	-	-	1
	Female (nos.)	-	-	-	-	-	-	-	-	-	-	-	-	-	-	1	-	-	1
Sediment	Mean (*X*φ)	2.5	2.4	2.4	2.0	2.2	2.1	2.0	2.0	1.6	2.4	2.6	2.3	2.2	2.2	2.3	2.2	2.2	2.2
	Sorting (*σ*φ)	0.8	0.7	0.8	0.9	0.9	1.0	0.9	1.1	1.2	0.5	0.5	0.5	0.6	0.6	0.6	0.7	0.7	0.8
	Skewness (*SK*φ)	-2.3	-2.3	-2.0	-1.6	-1.8	-1.5	-1.9	-1.4	-0.8	-2.4	-2.5	-2.2	-2.6	-2.6	-2.7	-3.1	-2.9	-2.5
	Kurtosis (*K*φ)	10.4	11.1	8.5	5.4	6.6	5.0	7.3	5.3	2.7	19.8	20.5	17.4	14.6	14.0	14.0	15.4	13.3	10.7
	Gravel (%)	0.7	0.6	0.7	1.6	1.3	2.1	2.3	3.0	3.2	0.3	0.3	0.3	1.3	0.9	0.6	1.3	1.7	2.1
	Sand (%)	97.9	98.2	97.8	97.9	98.1	97.5	97.4	96.3	95.7	99.2	99.2	99.4	98.5	98.7	99.0	98.6	98.0	97.5
	Silt & Clay (%)	1.4	1.2	1.5	0.5	0.6	0.5	0.4	0.8	1.1	0.5	0.5	0.3	0.3	0.3	0.4	0.1	0.2	0.3
	0.125 mm (%)	28.0	29.3	23.4	36.0	41.4	35.4	14.3	14.8	13.3	17.6	16.2	11.7	9.3	8.3	7.8	11.4	10.7	9.7
	0.180 mm (%)	49.3	47.4	45.1	26.0	27.6	28.0	47.5	43.7	34.0	66.1	62.9	66.7	63.4	65.7	70.6	71.5	69.3	64.8
	0.250 mm (%)	9.4	9.8	7.8	11.9	12.1	13.2	18.5	15.2	9.9	6.4	10.6	12.8	16.6	13.7	11.3	8.3	10.3	12.0
	Moisture depth (cm)	3.4	3.8	4.2	2.9	3.1	3.8	2.5	3.7	3.2	2.8	4.3	2.7	4.3	3.2	2.5	4.8	3.6	3.1
	Temperature (°C)	26.5	26.4	26.3	27.4	27.8	27.5	36.5	36.1	35.9	36.3	36.3	36.5	35.3	35.7	35.5	33.8	33.6	33.8
	pH	5.2	5.3	5.2	6.5	6.4	6.4	6.5	6.6	6.5	4.1	4.2	4.1	3.5	3.4	3.4	4.2	4.2	4.0
	Total Organic Carbon (%)	0.1	0.2	0.1	0.1	0.0	0.0	0.1	0.1	0.2	0.1	0.1	0.1	0.1	0.2	0.1	0.1	0.2	0.2
	Cd^{2+} (mg kg^{-1})	8.5	7.4	8.6	5.8	6.1	6.2	5.5	5.7	5.2	6.6	7.0	6.0	5.5	3.7	2.0	6.2	4.1	1.8
	Cr^{3+} (mg kg^{-1})	51.0	40.3	55.5	29.4	36.5	26.3	33.0	31.2	25.3	29.7	29.6	23.8	25.8	23.1	21.3	24.1	25.5	26.0
	Cu^{2+} (mg kg^{-1})	4.9	4.9	4.3	7.9	10.5	6.4	2.7	1.9	0.9	2.6	2.8	2.5	3.3	2.1	0.9	3.2	2.2	1.1
	Pb^{2+} (mg kg^{-1})	9.0	8.3	8.7	9.0	9.5	9.7	11.0	12.9	13.0	10.3	12.4	12.1	9.5	9.4	9.7	8.7	9.7	10.4
	Se^{2+} (mg kg^{-1})	16.1	15.4	14.9	10.6	9.3	13.3	14.0	14.3	12.6	17.0	18.4	16.4	6.2	6.3	6.6	14.8	15.6	15.9
	Zn^{2+} (mg kg^{-1})	31.2	29.7	28.8	25.2	25.3	28.5	21.6	20.7	17.0	20.7	22.3	19.7	15.6	14.9	14.8	17.8	18.8	19.2
Water	Temperature (°C)	24.3	24.4	24.5	26.6	26.6	26.6	30.4	30.2	30.3	29.8	29.9	29.7	30.3	30.4	30.4	30.0	30.1	30.1
	pH	6.8	6.9	7.1	5.6	5.8	6.2	6.7	6.2	5.9	6.9	6.5	6.3	8.1	7.7	7.5	8.8	8.4	8.2
	Salinity (‰)	0.5	0.9	1.5	1.8	2.8	3.2	8.2	9.4	10.2	4.1	5.6	6.4	11.1	12.6	13.3	14.9	15.3	17.0
	Dissolved Oxygen (mg l^{-1})	9.5	9.6	9.8	3.8	3.8	3.8	3.1	3.2	3.4	5.3	5.2	5.2	2.8	3.0	3.1	3.3	3.3	3.3
	NO_2^- (mg l^{-1})	0.0	0.0	0.0	0.0	0.0	0.0	0.1	0.1	0.1	0.0	0.0	0.0	0.0	0.0	0.0	0.0	0.0	0.0
	NO_3^- (mg l^{-1})	1.0	1.0	0.6	1.1	1.3	1.3	1.5	1.6	1.4	1.1	1.9	1.8	0.8	0.5	0.9	0.6	0.2	0.2
	PO_4^{3-} (mg l^{-1})	0.3	0.4	0.4	0.5	0.6	0.6	0.4	0.4	0.4	0.1	0.1	0.1	0.4	0.4	0.4	0.4	0.4	0.4
	S^{2-} (µg l^{-1})	56.7	55.0	55.3	62.7	66.0	72.0	31.3	28.7	29.7	68.0	66.0	63.3	14.3	12.7	14.0	27.3	29.0	26.3
	Chl-*a* (mg l^{-1})	0.1	0.1	0.1	0.0	0.0	0.0	0.1	0.1	0.1	0.0	0.0	0.0	0.1	0.1	0.1	0.1	0.1	0.1

'-' no sample availability during the investigation period

Table 5: Complete eco-biological observations from the *Tachypleus gigas* nesting sites (1-3) during the third series (2012) of new moon surveys at Pantai Balok

		2012																							
		May			June			July			August			September			October			November			December		
		S1	S2	S3	S1	S2	S3	S1	S2	S3	S1	S2	S3	S1	S2	S3	S1	S2	S3	S1	S2	S3	S1	S2	S3
Biology	Nest (nos.)	-	-	-	-	2	-	-	-	-	-	-	-	-	-	-	-	-	-	-	-	-	-	-	-
	Egg (nos.)	-	-	-	-	169	-	-	-	-	-	-	-	-	-	-	-	-	-	-	-	-	-	-	-
	Clutches (nos.)	-	-	-	-	4	-	-	-	-	-	-	-	-	-	-	-	-	-	-	-	-	-	-	-
	Male (nos.)	-	4	-	-	1	-	-	1	-	-	-	-	-	-	-	-	-	-	-	-	-	-	-	-
	Female (nos.)	-	1	1	-	-	-	-	-	-	-	-	-	-	-	-	-	-	-	-	-	-	-	-	-
Sediment	Mean ($X\varphi$)	2.4	2.3	2.6	2.6	2.6	2.7	2.5	2.4	2.4	2.3	2.5	2.5	2.6	2.6	2.4	2.6	2.6	2.4	2.5	2.6	2.2	2.5	2.5	2.6
	Sorting ($\sigma\varphi$)	0.8	0.8	0.6	0.5	0.6	0.5	0.7	0.8	0.7	0.9	0.8	0.7	0.5	0.6	0.7	0.6	0.6	0.7	0.5	0.5	1.0	0.6	0.7	0.4
	Skewness ($SK\varphi$)	-2.7	-2.2	-2.9	-2.5	-2.6	-2.5	-2.4	-2.6	-2.5	-2.5	-2.7	-2.7	-2.4	-2.7	-2.2	-2.7	-2.8	-2.5	-2.1	-2.2	-1.8	-2.3	-2.4	-1.2
	Kurtosis ($K\varphi$)	11.3	8.7	16.2	15.9	14.6	16.2	10.9	12.0	10.9	9.8	12.1	12.0	13.4	16.0	10.7	15.3	15.4	11.9	11.9	12.6	6.4	12.0	11.5	9.8
	Gravel (%)	2.5	1.9	0.8	0.2	0.6	0.2	0.7	1.4	0.6	2.6	1.6	0.5	0.4	0.4	0.9	0.2	0.3	0.7	0.1	0.2	2.2	0.2	1.0	0.1
	Sand (%)	97.1	97.7	98.4	99.5	98.5	98.8	98.9	98.1	99.1	97.1	98.1	99.2	99.1	98.6	97.9	99.4	98.6	98.9	99.7	98.7	97.5	99.4	98.2	98.9
	Silt & Clay (%)	0.3	0.4	0.8	0.4	0.9	1.0	0.4	0.5	0.3	0.3	0.3	0.3	0.5	1.0	1.1	0.4	1.2	0.3	0.2	1.1	0.3	0.4	0.8	1.0
	0.125 mm (%)	37.1	28.7	50.4	43.7	41.3	47.5	39.3	37.8	36.2	37.0	41.7	44.8	49.5	50.2	33.1	48.2	48.1	37.9	39.8	46.8	33.9	42.2	37.9	40.2
	0.180 mm (%)	38.6	41.4	29.3	37.1	34.8	30.3	37.2	36.5	43.3	40.4	36.3	33.5	31.0	29.8	37.0	30.8	27.7	39.6	40.7	30.4	32.3	37.8	34.5	39.6
	0.250 mm (%)	8.9	13.7	5.1	6.7	7.5	3.8	7.9	8.8	6.5	6.1	6.6	6.1	7.4	6.2	14.1	5.6	5.5	9.1	10.9	9.0	10.2	7.4	9.4	7.1
	Moisture depth (cm)	5.7	6.1	6.0	6.3	4.3	4.5	6.6	9.2	9.9	8.9	8.4	8.6	7.9	7.2	5.6	5.5	7.0	8.3	8.9	7.0	7.7	7.9	6.6	3.1
	Temperature (°C)	29.7	30.2	30.0	26.4	28.5	29.2	34.0	33.2	32.5	34.6	35.5	33.8	33.5	34.1	33.2	35.7	36.8	35.5	35.1	36.4	38.3	30.6	31.0	29.5
	pH	6.5	6.6	6.7	5.8	6.0	5.8	2.5	2.2	2.8	3.9	2.8	2.8	4.8	2.9	6.3	2.7	3.0	4.9	3.5	3.8	4.3	5.4	6.1	6.2
	Total Organic Carbon (%)	0.1	0.2	0.2	0.3	0.3	0.3	0.1	0.1	0.1	0.2	0.1	0.1	0.1	0.2	0.1	0.2	0.1	0.1	0.1	0.2	0.2	0.2	0.1	0.0
	Cd^{2+} (mg kg^{-1})	14.0	8.8	13.8	22.7	16.4	14.0	16.1	12.0	21.9	0.0	0.0	0.0	0.0	0.0	0.0	0.0	0.0	0.0	0.0	0.0	0.0	0.0	0.0	0.0
	Cr^{3+} (mg kg^{-1})	20.3	12.5	30.3	23.9	14.3	18.9	14.4	15.5	9.2	0.0	0.0	0.0	0.0	0.0	0.0	0.0	0.0	0.0	0.1	0.0	0.1	0.0	0.0	0.0
	Cu^{2+} (mg kg^{-1})	5.7	3.0	4.1	2.8	1.7	4.5	3.3	2.6	1.6	0.0	0.0	0.0	0.0	0.0	0.0	0.0	0.0	0.0	0.0	0.0	0.0	0.0	0.0	0.0
	Pb^{2+} (mg kg^{-1})	8.9	6.0	6.7	6.8	6.4	9.1	5.7	4.8	4.7	0.0	0.0	0.0	0.0	0.0	0.0	0.0	0.0	0.0	0.0	0.0	0.0	0.0	0.0	0.0
	Se^{2+} (mg kg^{-1})	19.7	25.8	31.7	24.6	25.3	16.2	17.3	25.4	28.0	0.0	0.0	0.0	0.0	0.0	0.0	0.0	0.0	0.0	0.0	0.0	0.0	0.0	0.0	0.0
	Zn^{2+} (mg kg^{-1})	22.0	16.3	20.0	21.7	17.4	22.6	15.9	14.3	13.7	0.0	0.0	0.0	0.0	0.0	0.0	0.0	0.0	0.0	0.0	0.0	0.0	0.0	0.0	0.0
Water	Temperature (°C)	29.8	29.7	29.8	29.5	29.7	29.5	29.1	28.9	31.2	31.4	31.1	30.6	29.7	30.2	30.0	30.4	30.5	32.2	29.8	29.4	29.8	29.1	28.1	28.8
	pH	6.0	6.2	6.4	8.3	8.3	8.4	7.0	7.5	8.1	7.7	8.0	7.9	7.8	7.7	7.5	8.5	8.1	7.5	7.1	7.0	7.0	7.5	7.6	7.0
	Salinity (‰)	36.0	35.9	36.8	33.2	33.0	33.1	27.4	28.4	33.5	31.5	26.9	26.2	15.3	16.6	19.5	13.2	15.2	20.7	3.8	3.9	5.0	14.5	14.6	16.2
	Dissolved Oxygen (mg l^{-1})	7.0	6.8	7.0	6.1	5.5	6.5	5.2	9.1	8.0	9.0	4.4	4.8	5.2	3.7	3.7	3.1	3.0	4.2	3.4	3.6	3.9	5.8	9.2	8.0
	NO_2^- (mg l^{-1})	0.0	0.0	0.0	0.0	0.0	0.0	0.0	0.0	0.0	0.0	0.0	0.0	0.1	0.1	0.0	0.1	0.1	0.0	0.1	0.1	0.1	0.0	0.0	0.0
	NO_3^- (mg l^{-1})	0.5	1.7	1.1	0.3	0.5	0.6	0.7	0.3	0.6	0.3	0.6	0.6	1.3	1.3	1.3	0.9	0.8	1.0	0.4	0.5	0.8	3.8	0.7	0.5
	PO_4^{3-} (mg l^{-1})	0.3	0.2	0.2	0.4	0.4	0.3	0.1	0.1	0.1	0.3	0.3	0.2	0.2	0.1	0.2	0.1	0.1	0.1	0.4	0.3	0.1	0.2	0.1	0.1
	S^{2-} (µg l^{-1})	2.0	3.0	1.3	41.0	33.3	9.0	5.3	7.3	7.7	67.3	24.7	13.0	47.3	40.3	54.7	24.3	23.0	36.0	23.7	18.7	20.7	20.0	60.3	10.0
	Chl-*a* (mg l^{-1})	0.2	0.3	0.2	0.5	0.6	0.4	0.5	0.4	0.6	0.9	0.3	0.4	0.6	0.7	0.4	0.3	0.5	0.4	0.6	0.6	0.9	0.4	0.6	0.2

'-' no sample availability during the investigation period

Table 6: Complete eco-biological observations from the *Tachypleus gigas* nesting sites (1-3) during the third series (2012) of full moon surveys at Pantai Balok

		2012																							
		May			June			July			August			September			October			November			December		
		S1	S2	S3	S1	S2	S3	S1	S2	S3	S1	S2	S3	S1	S2	S3	S1	S2	S3	S1	S2	S3	S1	S2	S3
Biology	Nest (nos.)	-	6	-	-	-	-	8	4	-	1	-	-	-	-	-	-	-	-	-	-	-	-	-	-
	Egg (nos.)	-	868	-	-	-	-	1074	613	-	254	-	-	-	-	-	-	-	-	-	-	-	-	-	-
	Clutches (nos.)	-	5	-	-	-	-	10	6		2	-	-	-	-	-	-	-	-	-	-	-	-	-	-
	Male (nos.)	1	2	-	-	2	-	1	3	1	-	-	-	-	-	-	-	-	-	-	-	-	-	-	-
	Female (nos.)	-	1	1	-	-	1	-	-	2	-	-	-	-	-	-	-	-	-	-	-	-	-	-	-
Sediment	Mean ($X\varphi$)	2.5	2.5	2.5	2.4	2.5	2.6	2.4	2.4	2.3	2.4	2.4	2.3	2.5	2.5	2.0	2.6	2.5	2.1	2.5	2.5	2.5	2.6	2.6	2.7
	Sorting ($\sigma\varphi$)	0.6	0.7	0.6	0.8	0.9	0.8	0.8	1.0	0.9	0.8	0.9	0.9	0.8	0.9	1.0	0.6	0.6	1.1	0.5	0.6	0.6	0.5	0.7	0.4
	Skewness ($SK\varphi$)	-2.7	-2.8	-2.8	-2.6	-2.6	-3.1	-2.7	-2.2	-2.2	-2.6	-2.4	-2.6	-2.6	-2.3	-1.2	-2.6	-2.4	-1.6	-1.9	-2.6	-1.7	-1.9	-2.5	-1.5
	Kurtosis ($K\varphi$)	14.9	14.0	15.7	11.3	10.8	14.6	11.7	8.4	7.8	11.2	9.8	10.4	10.9	9.3	4.3	12.6	10.8	4.7	12.0	14.1	9.0	15.0	12.6	12.6
	Gravel (%)	0.4	1.8	0.4	3.3	5.1	2.4	2.7	3.3	2.0	2.2	2.7	2.8	1.2	1.5	1.7	0.8	0.1	3.5	0.1	0.8	0.4	0.1	1.2	0.0
	Sand (%)	99.4	97.6	99.1	96.3	94.1	96.9	96.8	96.0	97.6	97.4	96.5	96.8	98.3	97.6	97.9	98.1	99.6	95.9	99.6	98.5	99.3	99.2	97.3	98.8
	Silt & Clay (%)	0.2	0.6	0.5	0.3	0.8	0.6	0.4	0.7	0.4	0.4	0.8	0.4	0.5	0.9	0.4	1.1	0.3	0.6	0.3	0.7	0.4	0.6	1.6	1.1
	0.125 mm (%)	40.6	37.5	39.4	34.9	41.9	46.6	39.2	34.6	36.0	35.4	37.2	34.0	44.5	44.0	24.4	48.3	46.3	34.8	42.3	40.3	36.8	41.1	36.7	45.9
	0.180 mm (%)	40.8	44.2	41.1	40.0	30.1	30.3	36.1	32.9	37.0	40.3	33.2	41.4	29.8	25.2	29.9	28.5	35.5	29.4	41.6	37.3	40.1	37.8	32.3	34.7
	0.250 mm (%)	7.7	7.5	7.6	10.5	5.1	4.2	7.8	9.3	7.2	8.2	7.9	9.1	8.5	9.1	16.7	6.8	6.5	9.5	9.0	10.1	12.2	5.7	7.5	2.9
	Moisture depth (cm)	6.0	6.1	6.8	4.9	5.0	4.4	7.1	6.4	7.5	6.3	6.4	9.1	8.8	8.8	6.4	5.4	5.2	6.7	5.3	6.1	5.8	8.6	8.6	8.3
	Temperature (°C)	30.0	28.1	28.9	31.7	32.8	32.3	28.6	32.4	33.5	33.8	32.5	31.4	34.0	35.2	36.1	35.2	36.2	36.7	36.0	36.3	36.5	29.3	28.1	29.1
	pH	5.9	5.8	6.1	6.8	6.5	6.8	4.0	5.4	4.6	3.0	3.8	2.8	3.2	5.3	6.5	2.8	4.8	4.2	3.0	3.9	4.1	4.2	5.7	5.8
	Total Organic Carbon (%)	0.1	0.2	0.2	0.2	0.2	0.1	0.2	0.1	0.0	0.2	0.1	0.1	0.0	0.1	0.2	0.0	0.0	0.0	0.2	0.2	0.1	0.1	0.2	0.1
	Cd^{2+} (mg kg^{-1})	15.7	13.3	2.7	18.5	16.4	11.9	26.7	16.8	14.7	9.3	11.1	15.5	0.0	0.0	0.0	0.0	0.0	0.0	0.0	0.0	0.0	0.0	0.0	0.0
	Cr^{3+} (mg kg^{-1})	28.7	17.0	17.9	37.7	10.7	13.5	22.4	43.7	10.7	11.0	9.6	15.6	0.0	0.0	0.0	0.0	0.0	0.0	0.0	0.0	0.0	0.0	0.0	0.0
	Cu^{2+} (mg kg^{-1})	2.9	2.2	2.4	2.2	2.2	1.6	2.1	3.7	2.1	5.9	2.5	4.0	0.0	0.0	0.0	0.0	0.0	0.0	0.0	0.0	0.0	0.0	0.0	0.0
	Pb^{2+} (mg kg^{-1})	6.4	7.8	5.1	5.6	7.4	4.9	4.1	4.7	4.8	8.3	8.3	10.8	0.0	0.0	0.0	0.0	0.0	0.0	0.0	0.0	0.0	0.0	0.0	0.0
	Se^{2+} (mg kg^{-1})	29.4	32.1	18.2	27.8	26.3	14.2	27.4	28.0	17.0	18.2	19.9	20.9	0.0	0.0	0.0	0.0	0.0	0.0	0.0	0.0	0.0	0.0	0.0	0.0
	Zn^{2+} (mg kg^{-1})	19.0	20.8	15.0	14.6	19.2	14.6	12.3	14.0	14.1	20.6	23.5	27.5	0.0	0.0	0.0	0.0	0.0	0.0	0.0	0.0	0.0	0.1	0.0	0.0
Water	Temperature (°C)	27.6	27.6	27.5	29.9	29.6	30.1	30.9	31.3	30.7	30.0	30.1	30.6	30.3	30.3	32.9	28.8	29.6	29.8	31.3	31.3	32.0	27.6	27.4	27.6
	pH	6.2	6.5	6.6	8.4	8.4	8.4	8.0	8.1	7.6	7.9	8.4	8.4	8.6	8.6	7.7	7.3	7.3	7.4	7.3	7.2	7.3	7.2	7.4	7.1
	Salinity (‰)	3.1	2.7	2.3	33.3	33.4	33.3	34.5	34.5	34.6	30.0	31.0	31.2	30.6	29.8	32.7	10.4	11.7	14.9	7.9	7.7	7.3	0.9	1.6	3.4
	Dissolved Oxygen (mg l^{-1})	2.8	3.2	4.1	5.3	5.1	5.6	6.8	5.4	6.7	7.8	5.8	9.1	5.1	4.4	4.7	4.4	5.6	4.2	4.2	4.4	4.7	4.9	6.0	3.4
	NO_2^- (mg l^{-1})	0.0	0.0	0.0	0.0	0.0	0.0	0.0	0.0	0.0	0.0	0.0	0.0	0.0	0.0	0.0	0.1	0.0	0.0	0.1	0.1	0.0	0.0	0.0	0.0
	NO_3^- (mg l^{-1})	0.2	0.3	0.3	0.7	1.4	0.3	0.7	0.3	0.6	3.4	0.6	0.7	0.7	0.9	1.1	0.3	0.1	0.6	0.4	0.4	0.5	0.4	0.3	0.1
	PO_4^{3-} (mg l^{-1})	0.1	0.2	0.1	0.0	0.0	0.1	0.1	0.1	0.1	0.0	0.1	0.1	0.0	0.4	0.1	0.1	0.1	0.2	0.5	0.4	0.3	0.2	0.3	0.2
	S^{2-} (µg l^{-1})	47.7	42.3	50.3	20.0	16.3	10.3	5.3	7.3	7.7	39.3	97.3	124.7	28.7	33.7	34.0	33.3	15.7	34.7	7.7	7.7	5.7	47.3	74.0	50.7
	Chl-*a* (mg l^{-1})	0.2	0.5	0.2	0.4	0.5	0.4	0.4	0.3	0.5	0.7	0.9	0.5	0.9	0.8	0.7	0.5	0.3	0.5	0.4	0.2	0.3	0.7	0.5	0.5

'-' no sample availability during the investigation period

Table 7: Complete eco-biological observations from the *Tachypleus gigas* nesting sites (1-3) during the third series (2013) of new moon surveys at Pantai Balok

		2013														
		January			February			March			April			May		
		S1	S2	S3	S1	S2	S3	S1	S2	S3	S1	S2	S3	S1	S2	S3
Biology	Nest (nos.)	-	-	-	-	2	-	4	-	-	-	-	-	-	-	-
	Egg (nos.)	-	-	-	-	47	-	418	-	-	-	-	-	-	-	-
	Clutches (nos.)	-	-	-	-	2	-	6	-	-	-	-	-	-	-	-
	Male (nos.)	-	-	-	-	-	-	-	-	-	-	-	-	-	-	-
	Female (nos.)	-	-	-	-	-	-	-	-	-	-	-	-	1	-	-
Sediment	Mean ($X\varphi$)	2.6	2.6	2.6	2.6	2.6	2.3	2.5	2.5	2.5	2.5	2.6	2.4	2.5	2.5	2.7
	Sorting ($\sigma\varphi$)	0.5	0.6	0.5	0.5	0.7	0.6	0.6	0.7	0.5	0.6	0.7	0.5	0.5	0.6	0.5
	Skewness ($SK\varphi$)	-2.0	-2.2	-2.0	-2.4	-2.4	-2.2	-2.2	-2.4	-1.5	-2.5	-2.6	-1.7	-2.0	-2.4	-2.3
	Kurtosis ($K\varphi$)	15.1	12.0	14.4	15.0	11.5	11.3	12.6	11.4	9.4	14.1	13.0	11.9	12.3	13.5	16.8
	Gravel (%)	0.3	0.6	0.4	0.3	1.9	2.0	0.2	1.0	0.2	0.4	0.8	0.4	0.2	0.7	0.3
	Sand (%)	99.1	98.4	99.1	99.1	97.1	97.8	99.2	98.1	99.3	99.2	97.5	99.4	99.0	98.6	99.3
	Silt & Clay (%)	0.7	1.0	0.4	0.6	1.0	0.3	0.6	0.9	0.5	0.4	1.7	0.2	0.8	0.6	0.4
	0.125 mm (%)	35.1	34.6	39.6	39.8	39.8	25.9	37.6	33.7	32.2	37.1	39.1	26.1	35.9	35.6	41.0
	0.180 mm (%)	40.5	34.0	43.5	38.4	32.2	47.7	39.0	33.4	43.0	40.7	28.0	53.0	41.0	40.5	33.4
	0.250 mm (%)	8.8	7.9	6.5	7.3	7.1	15.3	9.1	10.0	14.5	9.3	5.4	14.2	11.2	10.7	5.8
	Moisture depth (cm)	1.7	2.3	2.9	3.7	4.4	3.2	6.6	6.6	6.8	9.9	9.8	9.7	9.0	8.7	8.8
	Temperature (°C)	30.5	29.9	28.3	34.0	33.3	32.7	31.5	31.9	31.3	36.2	36.8	37.3	38.5	36.3	37.0
	pH	7.3	7.4	6.6	6.2	6.4	6.3	4.8	4.8	4.3	3.1	3.5	3.3	3.5	4.2	5.0
	Total Organic Carbon (%)	0.1	0.1	0.1	0.2	0.1	0.1	0.1	0.1	0.1	0.1	0.1	0.1	0.0	0.0	0.1
	Cd^{2+} (mg kg^{-1})	0.0	0.0	0.0	2.3	8.9	10.4	7.3	11.0	1.0	0.0	0.0	0.0	9.6	5.7	39.8
	Cr^{3+} (mg kg^{-1})	0.0	0.0	0.0	28.8	25.1	14.8	13.1	13.3	16.2	0.0	0.0	0.0	38.0	16.4	26.4
	Cu^{2+} (mg kg^{-1})	0.0	0.0	0.0	4.3	5.0	2.1	3.1	4.9	2.9	0.0	0.0	0.0	9.8	5.4	4.9
	Pb^{2+} (mg kg^{-1})	0.0	0.0	0.0	27.7	26.8	21.4	24.8	15.1	37.8	0.0	0.0	0.0	21.0	23.1	37.1
	Se^{2+} (mg kg^{-1})	0.0	0.0	0.0	15.5	17.0	18.9	29.0	15.2	25.1	0.0	0.0	0.0	30.2	26.8	40.2
	Zn^{2+} (mg kg^{-1})	0.0	0.0	0.0	31.5	36.5	23.3	28.1	25.8	29.3	0.0	0.0	0.0	30.5	31.4	46.2
Water	Temperature (°C)	27.5	27.5	27.7	30.3	29.6	30.0	29.4	29.6	29.5	30.8	31.4	32.1	30.5	31.4	31.6
	pH	7.7	7.8	7.2	8.1	8.1	7.6	8.1	8.1	7.8	8.6	8.5	7.0	8.7	8.6	8.0
	Salinity (‰)	5.3	6.0	8.2	4.1	4.4	5.4	12.4	12.6	13.9	25.0	26.5	29.2	22.0	23.2	26.2
	Dissolved Oxygen (mg l^{-1})	8.4	9.5	7.5	7.9	10.8	3.1	6.6	9.6	6.3	5.1	9.3	6.8	6.2	10.0	10.1
	NO_2^- (mg l^{-1})	0.0	0.0	0.0	0.0	0.0	0.0	0.0	0.0	0.0	0.0	0.0	0.0	0.2	0.2	0.1
	NO_3^- (mg l^{-1})	0.7	1.3	2.3	1.4	1.0	0.8	0.9	1.6	1.1	0.7	0.9	2.5	1.9	1.9	0.5
	PO_4^{3-} (mg l^{-1})	0.1	0.0	0.0	0.2	0.2	0.1	0.3	0.5	0.3	0.1	0.2	0.1	0.3	0.3	0.2
	S^{2-} (µg l^{-1})	14.7	37.0	25.3	48.0	50.3	54.3	25.3	40.3	11.0	28.7	32.0	18.0	13.0	27.3	18.7
	Chl-*a* (mg l^{-1})	0.6	0.4	1.0	0.6	0.5	0.5	0.4	0.3	0.2	0.7	0.8	1.0	0.5	0.6	0.5

'-' no sample availability during the investigation period

Table 8: Complete eco-biological observations from the *Tachypleus gigas* nesting sites (1-3) during the third series (2013) of full moon surveys at Pantai Balok

		2013														
		January			February			March			April			May		
		S1	S2	S3	S1	S2	S3	S1	S2	S3	S1	S2	S3	S1	S2	S3
Biology	Nest (nos.)	-	-	-	2	-	-	-	1	1	-	1	-	-	-	-
	Egg (nos.)	-	-	-	80	-	-	-	108	314	-	32	-	-	-	-
	Clutches (nos.)	-	-	-	3	-	-	-	1	3	-	1	-	-	-	-
	Male (nos.)	-	-	-	1	-	-	-	-	-	-	1	1	-	-	-
	Female (nos.)	-	-	-	-	-	-	-	-	-	-	1	-	-	-	-
Sediment	Mean (Xφ)	2.5	2.6	2.6	2.5	2.4	2.5	2.4	2.4	2.4	2.5	2.6	2.6	2.6	2.5	2.6
	Sorting (σφ)	0.5	0.6	0.5	0.6	0.7	0.4	0.7	0.8	0.6	0.5	0.6	0.4	0.5	0.6	0.5
	Skewness (SKφ)	-1.9	-2.4	-2.0	-2.0	-1.8	-1.1	-2.2	-2.3	-2.2	-2.1	-2.3	-1.8	-1.9	-2.1	-1.3
	Kurtosis (Kφ)	12.1	12.5	14.1	10.7	8.3	8.6	9.9	10.4	12.3	13.3	12.6	13.8	11.6	10.8	7.3
	Gravel (%)	0.4	0.6	0.2	0.4	0.6	0.2	0.7	1.8	0.7	0.4	0.5	0.2	0.2	0.9	0.0
	Sand (%)	98.8	98.4	99.4	99.1	98.3	99.4	98.5	97.3	99.1	99.2	97.9	99.4	99.1	97.8	99.4
	Silt & Clay (%)	0.7	1.0	0.4	0.5	1.2	0.3	0.8	0.9	0.3	0.5	1.7	0.4	0.6	1.3	0.6
	0.125 mm (%)	35.2	37.9	43.5	29.9	28.5	29.5	37.2	35.4	26.7	39.9	37.7	39.3	39.9	38.3	37.9
	0.180 mm (%)	39.8	32.9	38.1	39.8	34.5	50.3	35.7	35.2	49.6	39.8	32.1	44.8	37.3	33.5	37.6
	0.250 mm (%)	10.7	6.4	6.1	11.3	13.0	10.6	8.6	9.3	13.6	9.0	8.4	5.5	9.1	9.4	9.8
	Moisture depth (cm)	1.1	1.5	1.4	8.4	8.4	7.4	6.2	6.2	6.3	10.1	10.2	10.6	8.5	8.6	7.7
	Temperature (°C)	31.6	31.2	32.5	30.5	28.8	29.4	33.5	33.8	33.7	39.1	37.3	36.6	31.4	31.8	29.5
	pH	6.8	6.8	7.2	5.6	5.6	5.8	4.9	4.9	4.9	2.8	3.2	4.0	4.8	4.2	5.0
	Total Organic Carbon (%)	0.1	0.1	0.1	0.1	0.1	0.1	0.1	0.1	0.1	0.1	0.0	0.0	0.0	0.1	0.1
	Cd^{2+} (mg kg^{-1})	0.0	0.0	0.0	9.9	15.4	8.0	20.0	16.1	8.1	21.7	9.6	13.3	0.0	0.0	0.0
	Cr^{3+} (mg kg^{-1})	0.0	0.0	0.0	13.6	12.2	13.8	33.3	21.6	29.0	16.0	29.2	15.9	0.0	0.0	0.0
	Cu^{2+} (mg kg^{-1})	0.0	0.0	0.0	2.4	4.3	2.9	10.1	3.4	7.2	19.0	7.1	14.7	0.0	0.0	0.0
	Pb^{2+} (mg kg^{-1})	0.0	0.0	0.0	23.3	12.7	19.1	20.7	12.9	16.2	20.7	13.5	16.8	0.0	0.0	0.0
	Se^{2+} (mg kg^{-1})	0.0	0.0	0.0	15.5	15.2	28.0	36.6	23.4	10.1	21.3	28.0	19.7	0.0	0.0	0.0
	Zn^{2+} (mg kg^{-1})	0.0	0.0	0.0	23.8	23.2	23.6	41.6	28.9	30.0	26.8	26.3	27.3	0.0	0.0	0.0
Water	Temperature (°C)	29.8	28.9	31.3	28.5	28.7	27.7	30.8	31.1	30.9	32.8	31.6	30.6	30.0	28.7	29.9
	pH	8.0	8.1	7.3	7.9	7.7	7.4	8.6	8.7	8.7	8.2	7.9	7.4	8.3	8.0	7.3
	Salinity (‰)	9.9	9.4	10.1	3.7	3.9	5.4	20.9	21.7	21.7	16.9	15.0	16.7	23.9	24.6	27.3
	Dissolved Oxygen (mg l^{-1})	5.1	4.3	7.2	7.6	5.0	7.9	5.3	6.4	4.5	4.6	6.0	5.5	5.1	5.9	5.9
	NO_2^- (mg l^{-1})	0.0	0.0	0.0	0.1	0.0	0.0	0.0	0.0	0.0	0.1	0.1	0.1	0.0	0.0	0.0
	NO_3^- (mg l^{-1})	0.3	0.3	0.3	0.4	0.6	0.7	2.9	1.7	2.4	0.6	0.7	0.9	1.6	1.5	1.2
	PO_4^{3-} (mg l^{-1})	0.6	0.2	0.1	0.2	0.2	0.1	0.2	0.2	0.1	0.2	0.2	0.2	0.6	0.2	0.6
	S^{2-} (µg l^{-1})	29.0	9.0	16.7	23.3	22.0	66.7	0.0	4.7	5.0	58.7	35.7	22.0	14.7	5.0	3.3
	Chl-*a* (mg l^{-1})	0.7	0.4	0.6	0.6	0.9	0.5	0.4	0.6	0.5	1.0	1.2	1.0	0.3	0.2	0.2

'-' no sample availability during the investigation period
